# Instability of endosperm development in amphiploids and their parental species in the genus *Avena* L.

**DOI:** 10.1007/s00299-018-2301-x

**Published:** 2018-05-22

**Authors:** Paulina Tomaszewska, Romuald Kosina

**Affiliations:** 1 Institute of Experimental Biology, University of Wroclaw, Kanonia 6/8, 50-328 Wroclaw, Poland; 2 Institute of Environmental Biology, University of Wroclaw, Przybyszewskiego 63, 51-148 Wroclaw, Poland

**Keywords:** Amphiploids, *Avena*, Cytogenetics, Endosperm development, Somatic crossing-over, Species

## Abstract

**Key message:**

The development of oat endosperm is modified by chromatin and nuclei elimination, intrusive growth of cell walls, and polyploidisation of cell clones. The last event is correlated with somatic crossing-over.

**Abstract:**

Grass endosperm is a variable tissue in terms of its cytogenetics and development. Free-nuclear syncytium and starchy and aleurone endosperm were the main focus of the research. These were studied in oat amphiploids (4*x*, 6*x*, and 8*x*) and parental species (2*x*, 4*x*, and 6*x*). What the levels of cytogenetic disorders and developmental anomalies in species versus hybrids are, and, what the factors are determining phenotypes of both tissue components, are open questions for oats. Chromosome bridges and micronuclei are the main cytogenetic disorders showing the elimination of parts of genomes. Bridges are formed by the AT-heterochromatin-rich and -free ends of chromosomes. In the starchy tissue, various sectors are separated structurally due to the elongation or intrusive growth of aleurone cells. The development of the aleurone layer is highly disturbed locally due to the amplification of aleurone cell divisions. Changes related to their structure and metabolism occur in the aleurone cells, for example, clones of small versus large aleurone cells. Somatic crossing-over (SCO) is expressed in clones of large polyploidised cells (*r* = 0.80***), giving rise to new aleurone phenotypes. The multivariate description of the endosperm instability showed that endospermal disorders were more frequent in amphiploids than in the oat species. *Avena strigosa* and the amphiploid *A. fatua* × *A. sterilis* appeared to be extreme units in an ordination space. Nuclear DNA elimination, periclinal and multidirectional cytokineses, polyploidisation, intrusive growth, and SCO appeared to be important factors determining oat endospermal variations.

## Introduction

Some genetic and developmental phenomena are characteristics for endosperm tissue. Polyploidy and chromosomal disorders have been recognised to be common in the developing endosperm (Vijayaraghavan and Prabhakar 1984). These disorders and the subsequent elimination of nuclei in the apoptosis-like process create the background for the mosaic structure of endosperm in the embryo sac syncytium (Kosina 2007, 2016). McClintock’s important classical study on *Zea mays* provided strong data on the cytogenetics and development of endosperm (McClintock 1987). She evidenced transposons’ activity in the endosperm tissue and its clonal nature. Within a clone, developed from the outside towards the caryopsis inside, internal cells are large and polyploidised, while the external cells are younger and still undergoing division. Such a development indicates the direction of the endoreduplication gradient (Becraft and Gutierres-Marcos 2012). The endoreduplication and the metabolite storage potential decrease towards the outer parts of the caryopsis, and probably, both these processes are closely related. Such a clonal nature determines tissue patterns expressed during the endosperm development. Among cereals, endospermal syncytia, or tissues have been most extensively studied in wheat (Morrison et al. 1975) and barley (Bosnes et al. 1992). In maize, a further in-depth study using molecular methods provided new data (Becraft and Yi 2011). The *dek1, crinkly4*, and *sal1* genes were discovered to play a significant role in controlling endosperm development. In addition, Gruis et al. (2006) observed that the cells of the aleurone layer under the control of the genes *dek1* and *sal1* can also be developed inside the starchy endosperm. Their most important conclusion was that developmental fates of starchy and aleurone cells are interchangeable. Endosperm cytogenetics and development have also been analysed in the interploidy barley hybrids (Håkansson 1953), Triticale (Kaltsikes 1973), and *Triticum* × *Leymus* F_1_ progeny (Ivanovskaya 1983). However, there has been much less study into oat endosperm, especially that of the hybrid origin. Kosina and Tomaszewska (2011) provided data on variations in oat amphiploids endosperm, whereas Florek and Kosina (2017) showed new variability in the endosperm structure after demethylation of the *Avena barbata* × *A. sativa* ssp. *nuda* amphiploid genomes.

Especially in oats, some questions on endosperm are still unanswered; namely, concerning:

- the level of variations in terms of cytogenetics, growth, and tissue architecture expressed in oat species of varying ploidy levels versus their hybrid progeny;
- factor(s) important for the development of the aleurone phenotype.

In plant endosperms, many genes are imprinted and endoreduplication and programmed cell death (PCD) are common (Becraft and Gutierrez-Marcos 2012). The main events that are important for further endosperm development occur at its free-nuclear stage. Many anomalous nuclei are eliminated in a PCD-like process (Kosina 2016). Such a nuclear selection presents new possibilities for later karyokineses, cytokineses, and growth. In addition, Tomaszewska (2017) discovered that, in both, oat species and amphiploids, polyploidy of the endosperm is not at the triploid but diploid level. Thus, is the oat endosperm an apomictic autonomous or pseudogamous unit, according to Nogler’s classification (Nogler 1984)? That ploidy status is different from the *Polygonum* type of embryo sac, common in grasses, and it changes genomic interrelationships in the endosperm nuclei as well as genetic control of endosperm development.

## Materials and methods

### Plant material

Young endosperms, for cytogenetic analyses of the free-nuclear syncytium, as well as endosperm tissue from ripe caryopses of six oat amphiploids and their parental species were sampled from plants, that were cultivated on small plots or in pots in the grass collection (Wroclaw, SW Poland), maintained by R. Kosina. During the plot or pot experiments, plants were grown under the same soil-climatic conditions. Thus, the study material was treated as originating from a completely randomised one-way classification design. Oat accessions used in the study are listed in Table 1.

**Table 1:** Oat accessions of amphiploids and parental species used in the study

Amphiploid/parental species	Symbols in a diagram	Accession number	Donor	Origin	Ploidy level
*A. barbata* × *A. sativa* ssp. *nuda*	b/sn	CIav7903	NSGC	–	Octoploid
*A. eriantha* × *A. sativa*	e/s	PI458781	NSGC	–	Octoploid
*A. barbata* × *A. sativa*	b/s	CIav7901	NSGC	–	Hexaploid
*A. fatua* × *A. sterilis*	f/ste	CIav9367	NSGC	–	Hexaploid
*A. magna* × *A. longiglumis*	m/l	CIav9364	NSGC	–	Hexaploid
*A. abyssinica* × *A. strigosa*	a/str	CIav7423	NSGC	–	Tetraploid
*A. fatua* L	Af	–	R. Kosina	Poland	Hexaploid
*A. sterilis* L	Aste	PI311689	NSGC	Israel	hexaploid
*A. sativa* L	As	–	R. Kosina	Poland	Hexaploid
*A. barbata* Pott ex Link	Ab	AVE1938	BAZ	Spain	Tetraploid
*A. abyssinica* Hochst	Aa	PI331373	NSGC	Ethiopia	tetraploid
*A. magna* Murphy et Terrell	Am	1786	VIR	Morocco	Tetraploid
*A. strigosa* Schreb	Astr	51,624	BAZ	Belgium	Diploid
*A. longiglumis* Dur	Al	PI367389	NSGC	Portugal	Diploid
*A. eriantha* Dur	Ae	CIav9051	NSGC	England	Diploid

Bundesanstalt für Züchtungsforschung an Kulturpflanzen, Braunschweig, Germany (BAZ); National Small Grains Collection, Aberdeen, Idaho, USA (NSGC); Vavilov Institute of Plant Industry, St. Petersburg, Russia (VIR)

### Cytogenetic preparation

The analyses were made at the nuclear, syncytial stage of the endosperm. Endosperms were isolated from the young caryopses, and fixed in Carnoy fixative between 2 and 3 DAP (days after pollination). Cytogenetic data were obtained after DAPI staining (2 µg/ml) of squashed endosperm nuclei and mitoses, which were observed under an epifluorescence microscope (Olympus BX-60; Hamburg, Germany). Results were obtained for random samples, *n* = 30 endosperms for each accession.

### Endosperm preparation

Mature caryopses were fixed in FAA fixative (formalin:acetic acid:ethanol) for several days at room temperature and then rinsed three times in distilled water before preparation. The aleurone layer from the middle part of the caryopsis was manually isolated. The pericarp, testa, and starchy endosperm cells were removed using a microscalpel. Isolated aleurone layers were mounted in glycerol. Grains fixed in FAA fixative were cut transversely in the middle part of the caryopsis with the use of a freezing microtome. Tissue slides were sealed in glycerol. All caryopsis preparations (aleurone layer and cross-sections) were made for random samples, *n* = 60 caryopses for each accession.

### Microscopy

The research specimens were analysed in an Olympus BX-60 microscope (Hamburg, Germany) with the use of a UV filter for DAPI or tissue-own fluorescence, and in an Amplival polarising microscope (Carl Zeiss, Jena, Germany). Images were taken with an Olympus E-520 camera (Olympus Imaging Europa GMBH, Hamburg, Germany).

### Numerical taxonomy

Oat amphiploids and species treated as operational taxonomic units (OTUs) were described by multivariate data of eight characters of aleurone layer (see Table 2 for the characters). The matrix of average taxonomic distances between 15 OTUs was calculated. This matrix was transformed into a configuration matrix using the Kruskal’s non-metric multidimensional scaling method (nmMDS), and the latter matrix was applied to set OTUs in a minimum spanning tree (MST) in a three-dimensional (*x, y*, and *z*) ordination space. Numerical analyses were performed using the NTSYS software (Rohlf 1994).

**Table 2:** Frequency of various types of disorders observed in the aleurone layer of amphiploids and parental species

Amphiploids and parental species	Traits of disorder							
	IAC	LV	LG	CSC	LC	CLC	SC	SCO
*A. barbata* × *A. sativa* ssp. *nuda*	60.0	–	6.7	6.7	60.0	6.7	63.3	6.7
*A. eriantha* × *A. sativa*	86.7	–	26.7	6.7	46.7	16.7	43.3	–
*A. barbata* × *A. sativa*	73.3	–	13.3	3.3	40.0	20.0	46.7	–
*A. fatua* × *A. sterilis*	89.7	–	13.1	–	55.1	48.6	68.2	5.0
*A. magna* × *A. longiglumis*	81.8	–	3.0	–	63.6	24.2	57.6	–
*A. abyssinica* × *A. strigosa*	64.4	–	43.8	22.0	21.9	5.5	30.1	–
∑/*n* (for amphiploids)	**76.0**	**0**	**17.8**	**6.5**	**47.9**	**20.3**	**51.5**	**2.0**
*A. fatua*	20.0	–	16.7	–	16.7	10.0	26.7	–
*A. sterilis*	33.3	3.3	3.3	–	30.0	3.3	26.7	–
*A. sativa*	76.7	13.3	–	–	40.0	13.3	30.0	–
*A. barbata*	43.3	6.7	–	–	23.3	6.7	26.7	–
*A. abyssinica*	10.0	3.3	–	–	13.3	–	23.3	–
*A. magna*	36.7	–	6.7	–	43.3	16.7	10.0	–
*A. strigosa*	40.0	–	23.3	–	13.3	–	3.3	–
*A. longiglumis*	26.7	10.0	–	3.3	13.3	–	10.0	–
*A. eriantha*	40.0	–	–	–	6.7	–	–	–
∑/*n* (for species)	**36.3**	**4.1**	**5.5**	**0.4**	**22.2**	**5.6**	**17.4**	**0**

The values are in terms of percent

*IAC* ingrowth of aleurone cells, *LV* large vacuoles, *LG* large globoids, *CSC* clones of small cells, *LC* large cells, *CLC* clones of large cells, *SC* starchy cells, *SCO* somatic crossing-over, *∑/n* arithmetic averages (%) for the amphploid and species traits

## Results

In oats, the endosperm tissue variants mostly depend on growth relationships between both, starchy and aleurone components. Many different cellular phenotypes are also created on a variable genetic background.

### Cytogenetic disorders

**Fig. 1:**
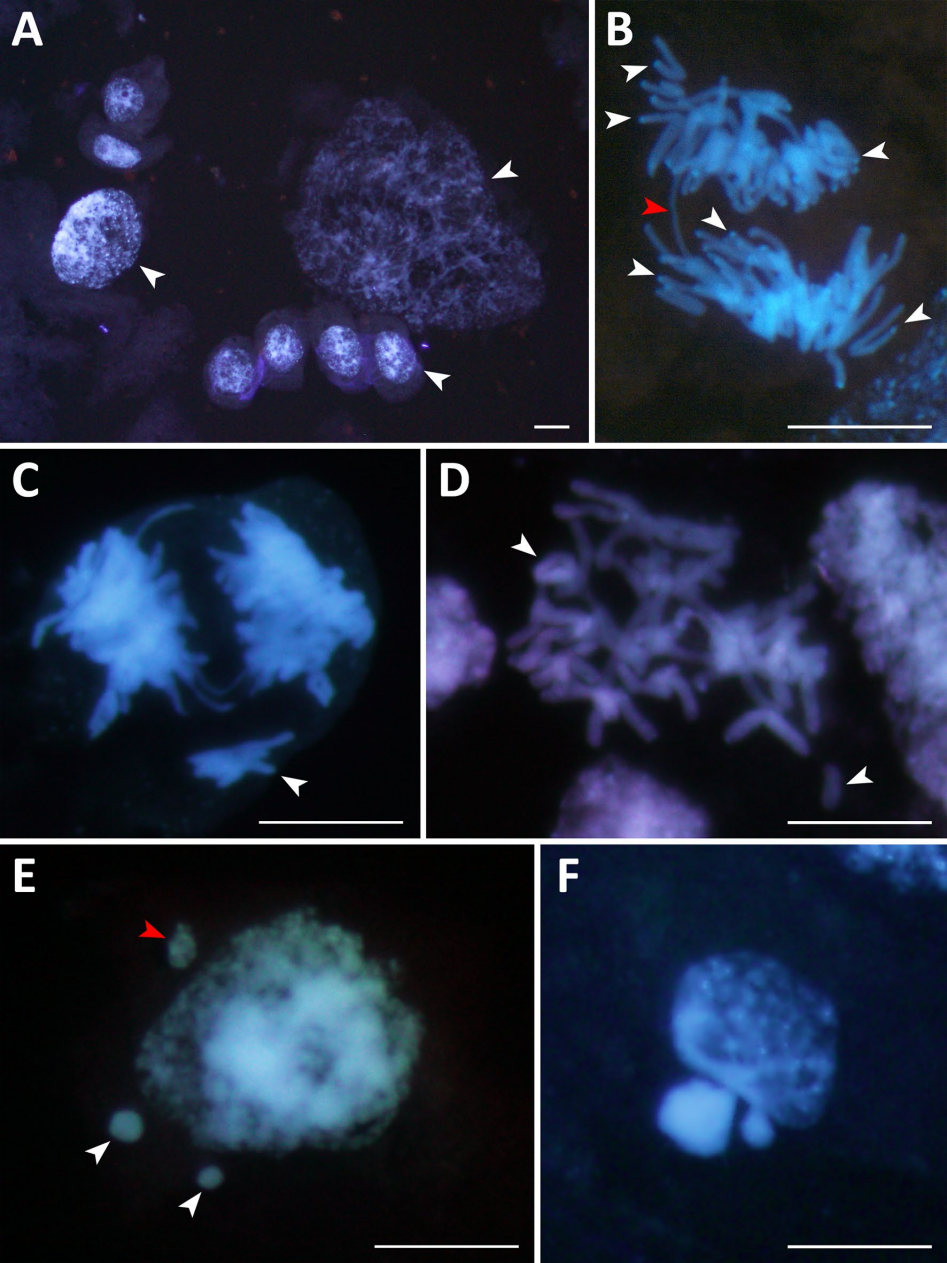
Examples of data on cytogenetic disorders in the oat nuclear endosperm. **a** Nuclei at various levels of ploidy (white arrowheads) in *A. longiglumis*; **b** a bridge (red arrowhead) in the amphiploid *A. abyssinica* × *A. strigosa*. Some chromosomes with telomeric AT-heterochromatin not creating bridges are shown by white arrowheads. **c** Bridges and a group of delayed chromosomes (white arrowhead) in the amphiploid *A. fatua* × *A. sterilis*; **d** ring and telocentric chromosome (white arrowheads) in the amphiploid *A. fatua* × *A. sterilis*; **e** micronuclei at various level of condensation in *A. sterilis* (red arrowhead for less and white for more condensed); **f** a nucleus with extruded condensed chromatin in *A. eriantha*. Scale bars 20 µm. (Color figure online)

The endosperm genetic background is changed during the earliest stage of development, i.e., its free-nuclear stage. Various cytogenetic disorders were noted for both types of oats: amphiploids and parental species. As a rule, endospermal nuclei present a broad range of polyploidy, seen as nuclei of various sizes (Fig. 1a). Highly polyploid nuclei were noted in 83% of amphiploids and 33% of parental species. The hyperploid metaphases occurred with a frequency of 1.42% in amphiploids versus 0.5% in species. The chromosomal bridges, telecentrics, and rings (Fig. 1b–d) support the conclusion that a breakage-fusion-bridge (BFB) cycle occurs in the free-nuclear syncytium. The frequency of bridges is similar in both amphiploids (11%) and species (13%). The bridges were not observed in *A. magna* × *A. longiglumis*, and *A. sativa*. Rings were formed in 50% of amphiploids versus 22% of parental species. In oat units, rings were formed at low frequency, 1.0–1.5%. Knobs of DAPI condensed chromatin (AT-heterochromatin) were detected on bridges in *A. barbata* × *A. sativa* ssp. *nuda, A. barbata* × *A. sativa*, and *A. eriantha* (Tomaszewska 2017). Such a positioning of DAPI knobs suggests that telomeric heterochromatin is involved in bridge formation. However, many bridges have no trace of heterochromatin. In addition, chromosomes with distinct AT-heterochromatin at telomeres do not form the bridge connections between telophase nuclei (Fig. 1b). The delayed single chromosomes or their groups (see Fig. 1c) were eliminated in the form of less or more condensed micronuclei (Fig. 1e). In both groups of oats, micronuclei were noted in every syncytial endosperm. However, on average, the number of micronuclei per cell is distinctly larger in amphiploids than in parental species (3.5 versus 1.9). Finally, during an apoptotic-like chromatin process is secreted from the nuclei as condensed bodies (Fig. 1f). On average, all observed cytogenetic anomalies reached a 61% frequency in amphiploids and 44% in parental species.

### Macrodisorders

**Fig. 2:**
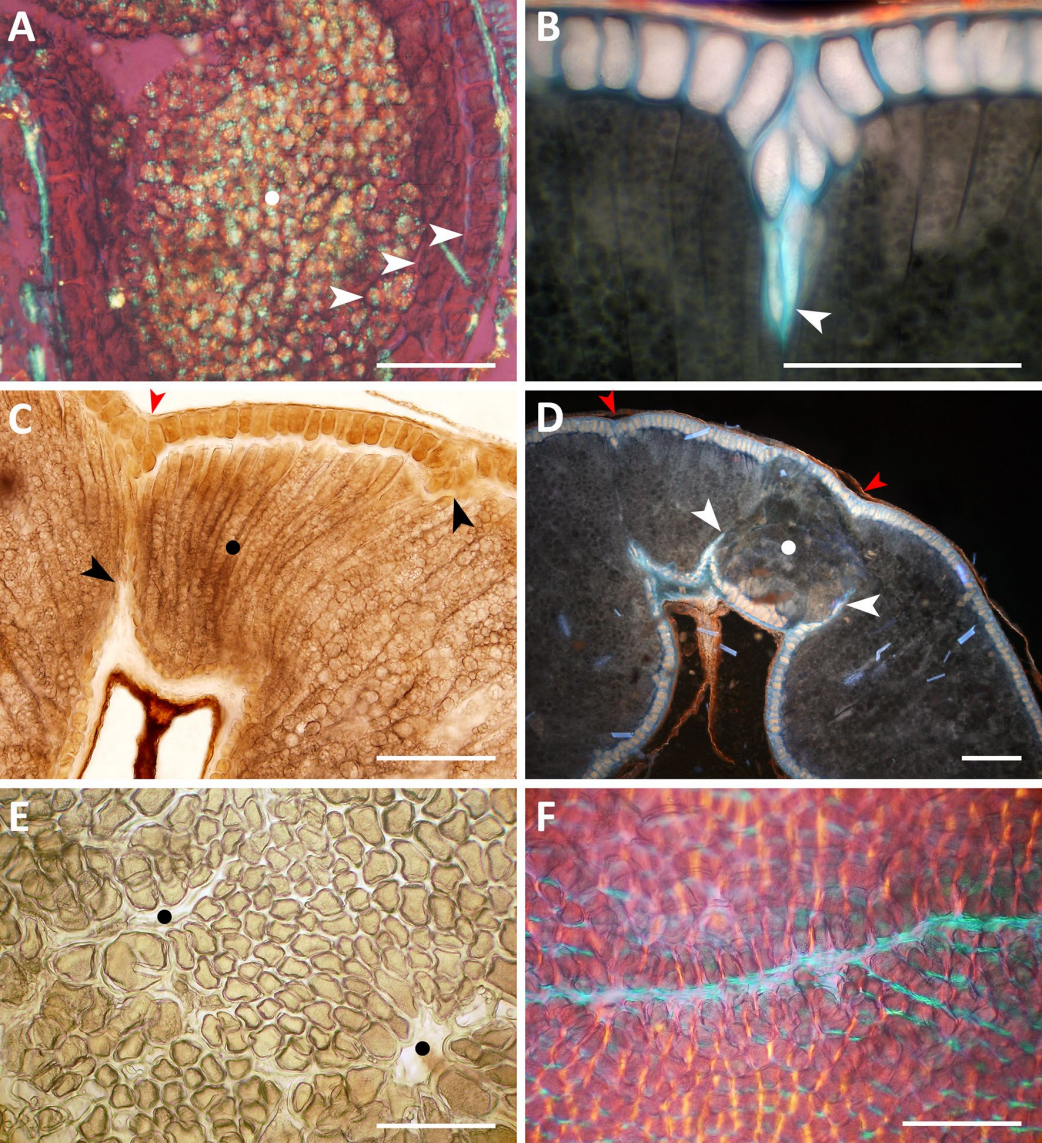
Examples of various types of developmental macrodisorders in the cellular endosperm. **a** An a-cellular starchy domain in the lateral part of the caryopsis (white dot) and adjacent to it starchy, subaleurone, and aleurone cellular parts (white arrowheads) in *A. longiglumis*; **b** intrusive ingrowths of aleurone cells into the starchy endosperm in the amphiploid *A. fatua* × *A. sterilis* (white arrowhead); **c** a domain with a distinct elongated growth of starchy cells (black dot) separated by aleurone cell ingrowths (black arrows) in the amphiploid *A. abyssinica* × *A. strigosa*, a concaved surface of the caryopsis is shown by a red arrowhead; **d** a high-protein domain (white dot) bounded by aleurone ingrowths (white arrowheads), concaved surface of caryopsis marked by red arrowheads in the amphiploid *A. fatua* × *A. sterilis*; **e** numerous disorders in the aleurone layer of the amphiploid *A. fatua* × *A. sterilis* (black dots in empty spaces); **f** a slit in the aleurone layer in the amphiploid *A. fatua* × *A. sterilis*. **a, c, e**, and **f** in a polarising microscope at differently crossed nicols; **b** and **d** in an epifluorescence microscope. **a**–**d** for caryopsis cross-sections; **e** and **f** for a surface-view of the aleurone layer. Scale bars 100 µm. (Color figure online)

**Fig. 3:**
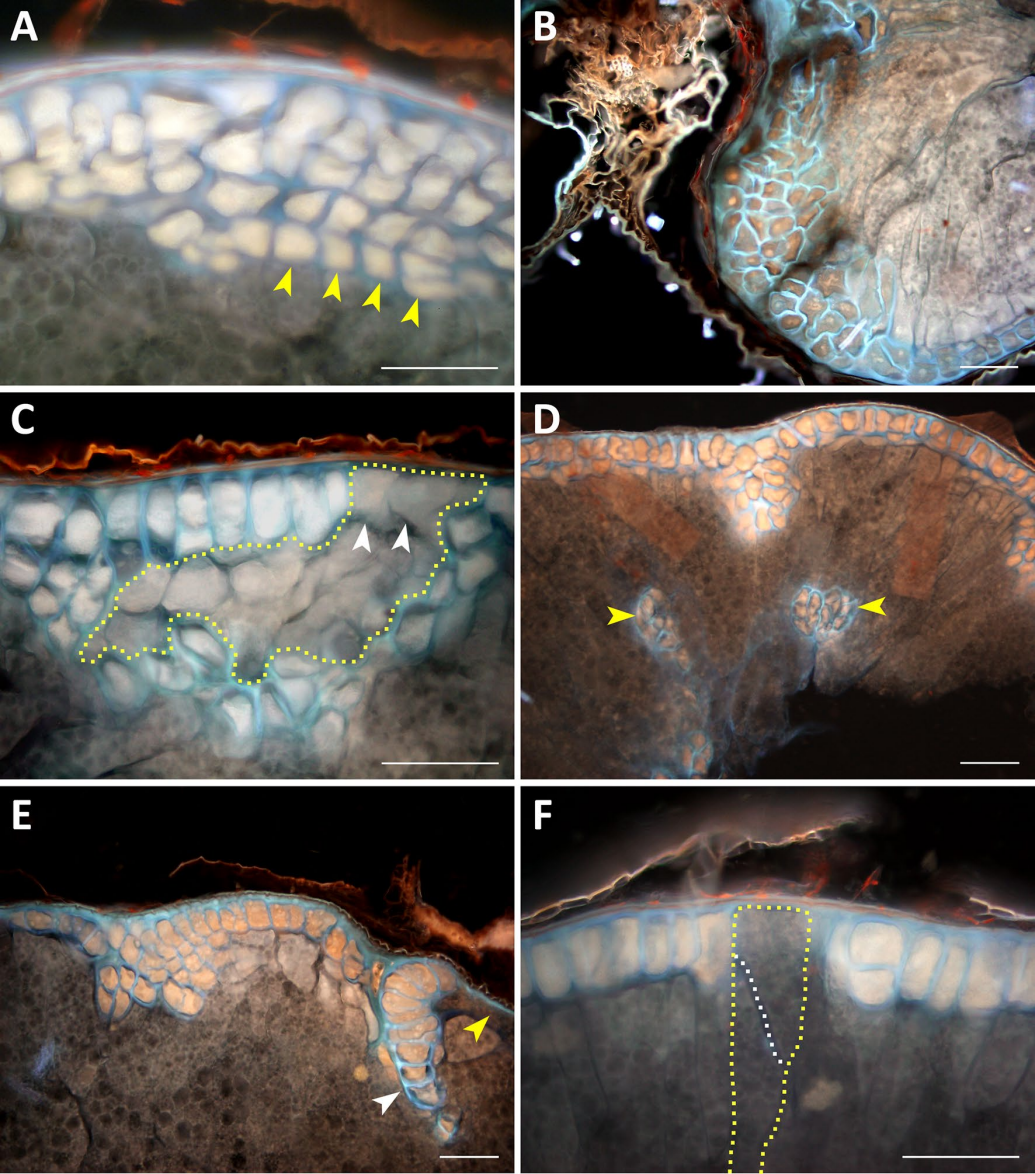
Examples of anomalous development of the aleurone layer. **a** Multilayered aleurone formed after clear periclinal divisions (yellow arrowheads) in the amphiploid *A. abyssinica* × *A. strigosa*; **b** a multilayered aleurone (blue walls) formed after periclinal and multidirectional divisions in *A. magna* × *A. longiglumis*; **c** a domain of the high-protein subaleurone cells (dotted yellow line) surrounded by aleurone cells in the amphiploid *A. eriantha* × *A. sativa*; the last periclinal divisions are shown by white arrowheads; **d** aleurone cells inside the starchy endosperm (yellow arrowheads) in the amphiploid *A. fatua* × *A. sterilis*; **e** aleurone layer with disturbed cell divisions in the amphiploid *A. fatua* × *A. sterilis*, a clone of large cells marked by a white arrowhead and an a-aleurone part by a yellow arrowhead; **f** two large starchy cells (dotted yellow outline) divided diagonally but not periclinally (dotted white line) differentiated within the aleurone layer in the amphiploid *A. fatua* × *A. sterilis*. **a**–**f** caryopsis cross-sections seen in an epifluorescence microscope. Scale bars 100 µm. (Color figure online)

**Fig. 4:**
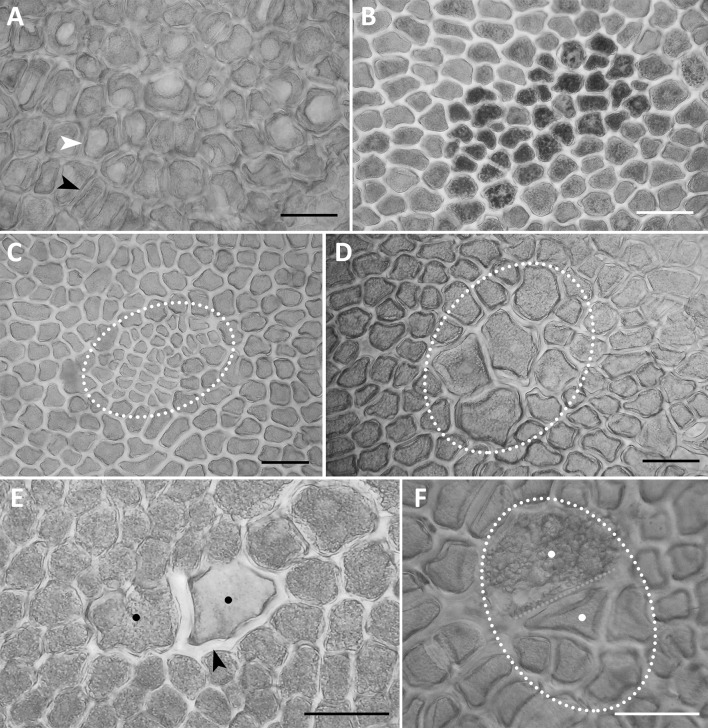
Examples of variability of an isolated aleurone layer (a surface view). **a** Vacuolar (white arrowhead) and a-vacuolar (black arrowhead) parts of the aleurone layer in *A. sativa*; **b** a group of dark aleurone cells with multiple globoids in the amphiploid *A. abyssinica* × *A. strigosa*; **c** a clone of small aleurone cells (outlined) with a short cell cycle in the amphiploid *A. barbata* × *A. sativa* ssp. *nuda*; **d** a clone of large aleurone cells (outlined) with a long cell cycle in the amphiploid *A. fatua* × *A. sterilis*; **e** two phenotypes (black dots) of polyploid sister aleurone cells after somatic crossing-over—the left cells with normal aleurone grains and cell wall, the right with a thick hemicellulosic wall (black arrow), and light aleurone grains in the amphiploid *A. abyssinica* × *A. strigosa*; **f** two sister cells (outlined and white dots) in the aleurone layer expressing two phenotypes; the lower cell is ‘aleurone’ and higher is ‘starchy’; after somatic crossing-over in the amphiploid *A. barbata* × *A. sativa* ssp. *nuda*. **a**–**f** documented in a polarising microscope at differently crossed nicols. Scale bars 100 µm

Examples of developmental events in endosperm tissue are presented in Figs. 2, 3, and 4. Most often, the adult caryopsis is filled with endospermal cells. These cells are stores of various metabolites, such as starch, protein, fat, and celluloses. In cases, when the rate of starch synthesis is high and cytokineses are delayed, the centre of a ripe caryopsis, between dorsal and ventral parts, is a-cellular and is filled with starch granules. Such a developmental event is not frequent. In *Avena longiglumis*, another example of the a-cellular endosperm is shown (Fig. 2a). Not the central but the lateral part of the caryopsis is a-cellular and filled with large composite starch granules. Some layers of aleurone, subaleurone and starchy cells surround this a-cellular domain. Such a development is anomalous and rare. Ingrowths of aleurone cells into starchy endosperm (Fig. 2b) were observed in all amphiploids and parental species (Table 2). On average, the ingrowths were found to be more frequent in amphiploids (76%) than in parental species (ca 36%). These ingrowths are realised by the anticlinal elongation of aleurone cells and/or the intrusive growth of their walls (Fig. 2b). The aleurone cells can penetrate the starchy endosperm from the dorsal and ventral parts of the caryopsis; they join and separate some areas of the starchy tissue. The separated starchy units often differ from each other in terms of development and metabolism (Fig. 2c, d). Just above the endosperm cavity, the starchy domain is composed of cells showing elongation or intrusive growth (Fig. 2c) or a callus-like body with mainly proteinaceous cells which can develop there (Fig. 2d). Slits and endosperm losses seen on the surface of an isolated aleurone layer are correlated with more voluminous ingrowths (Fig. 2e, f). The aleurone and nucellar ingrowths are seen as depressions on the surface of the caryopsis (Fig. 2c, d).

### Disorders of the aleurone layer

Caryopsis cross-sections show that the development of the aleurone layer is highly variable (Fig. 3). In some areas of the section, the layer is multicellular and is formed by regular periclinal cytokineses (Fig. 3a), or it develops in the form of a callus-like body due to many multidirectional cell divisions (Fig. 3b). A highly proteinaceous (HP) subaleurone layer, in the form of an irregular multicellular clone, can develop in the aleurone layer (Fig. 3c). Despite the last periclinal cytokinesis occurring in this unit (see arrows), which should induce the expression of the aleurone phenotype, its protein phenotype is not “aleurone” but “subaleurone”.

The development of the aleurone phenotype within the starchy endosperm was rarely noted (Fig. 3d). In the aleurone layer, clones of the aleurone cells can develop separately (see the clone in Fig. 3e). This clone is adjacent to the area that is free of aleurone cells. Such clones are composed of large, polyploidised cells. The presented examples show some aleurone layer mosaic. This mosaic was supplemented by the development of a large starchy cell within the aleurone layer (Fig. 3f). It should be noted that this cell was not subjected to periclinal division. Its sister cell was formed after a diagonal cytokinesis (see the marks in the figure). These starchy cells are older and distinctly larger than the aleurone cells and possibly polyploidised.

Additional data on the developmental disorders of aleurone cells are provided using the aerial view of the isolated aleurone layer observations (Fig. 4). Two morphotypes depending on the vacuole development may be recognised: one is without microscopically visible vacuoles; the second expresses large vacuoles (Fig. 4a). The “vacuole” morphotype is expressed in the form of large spots composed of tens or hundreds of cells, and was observed in species, but not in amphiploids (Table 2). Single aleurone cells or multicellular spots also develop in the form of dark units due to the development of many larger globoids in the aleurone grains (Fig. 4b). Other changed aleurone cell clones are groups of small versus large cells, the dimensions of which differ distinctly from normal cells. This difference in relation to the cell size is due to either 1–2 additional cytokineses or their lack (Fig. 4c, d). Such cell clones have a shorter or longer cell cycle. The occurrence of a single large or small aleurone cell (LC and SC in Table 2) was a frequent event in amphiploids and they were 2–3 times less frequent in the parental species. The two different phenotypes expressed in the sister aleurone cells demonstrate that somatic crossing-over (SCO) occurred during mitosis in their mother cell. Two examples of SCO are presented in Fig. 4e, f. The first relates to the difference in protein and cellulose synthesis in both the cells. The changed cell stores lighter protein due to the defect in globoid synthesis and develops a thicker hemicellulosic wall. The second case relates to the difference between the stored materials; synthesis of protein versus starch in two sister aleurone cells. SCO was noted in two amphiploids (Table 2), on average in 2.0% of individuals.

**Fig. 5:**
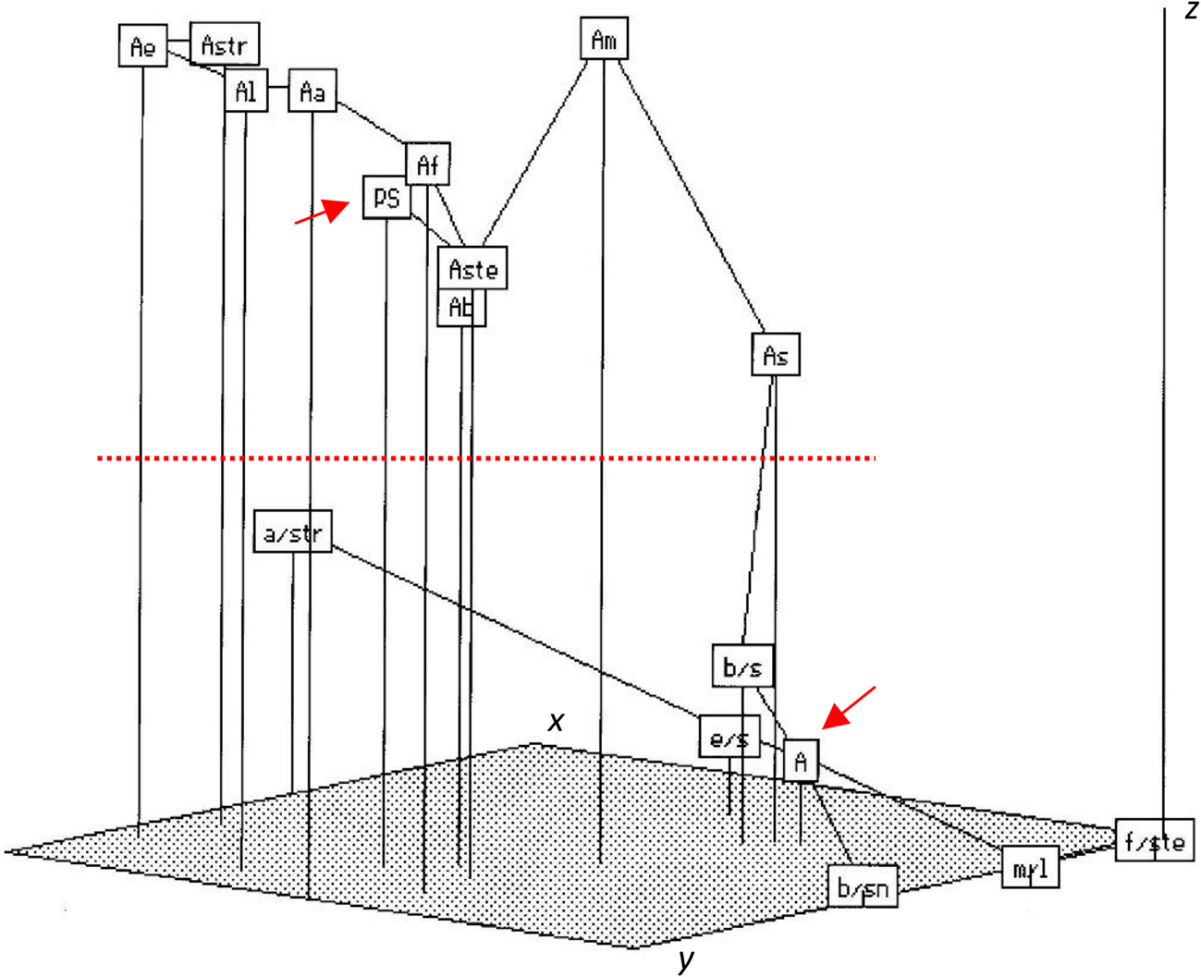
Minimum spanning tree of amphiploids and parental species (OTUs) of the genus *Avena* in an ordination space (*x-, y-*, and *z*-axes) and created by application of Kruskal’s non-metric multidimensional scaling method (Rohlf 1994). OTUs were described by eight disorder traits of the aleurone layer {red arrows for an average for parental species (PS) and for amphiploids (A); other abbreviations in the accession list; a red dotted line separates species and amphiploids}. (Color figure online)

Some types of endospermal disorders, such as ingrowths of aleurone cells into the starchy tissue, large aleurone cells, and starchy cells in the aleurone layer were more common and were noted in both amphiploids and parental species. Clones of small aleurone cells are rare in species; SCO was observed only in amphiploids (see Table 2). On average, the disorders occurred with a higher frequency in amphiploids when compared with species. These data are summarised in Fig. 5. In an ordination space, amphiploids and parental species can be distinctly discriminated. *Avena abyssinica* × *A. strigosa* (a/str) is distant from other amphiploids when looking at its position along the *x-* and *y*-ordination-axes, but it is close to them in therms of *z*-axis value.

On average, amphiploids (A) and parental species (PS) are well distinguished (Fig. 5). The highest level of disorders was expressed in the amphiploid *A. fatua* × *A. sterilis* (f/ste), whereas the lowest was noted in *A. eriantha* (Ae), and, in the diagram, this relationship is related to the lowest versus the highest of *x-, y-*, and *z*-ordination-axis values, respectively.

For all pairs of traits describing disorders in endosperm, Pearson’s coefficients of correlation were calculated. Two coefficients were significant at *α* = 0.001: SC (starch cell) versus LC (large cell), *r* = 0.84; and SCO (somatic crossing-over) versus CLC (clones of large cells), *r* = 0.80. These correlations indicate that, in a pool of large (polyploidised) cells in the aleurone layer, most such cells are starch cells and that somatic crossing-over occurs most often within the clones of large cells.

## Discussion

### Cytogenetics

Cytogenetic anomalies in the free-nuclear syncytium were studied in Triticale, the first amphiploid that is important for human nutrition (Kaltsikes 1973). Extremely large polyploid nuclei, multiple bridges, chromatin connections between neighbouring anaphases, and multiple fusions of nuclei were most often observed. Vijayaraghavan and Prabhakar (1984) pointed out that, in degenerating endosperm, highly condensed nuclei are often connected by thick chromatin links. Such nuclei were evidenced in our oat material (Tomaszewska 2017). In *Triticum* × *Elymus* F_1_ hybrids, the young endosperms was poorly filled by nuclei, starch, and later by cells. Initially, a few separate groups of small or huge nuclei were scattered along the syncytium wall (Ivanovskaya 1983). Kosina (1996) detected such nuclear groups in other grasses and described them as subsyncytial units. In Triticale, Bennett (1977) proved a positive correlation between the frequency of anomalous nuclei in the syncytial endosperm and developmental anomalies in ripe caryopses as well as between the amount of rye telomeric heterochromatin and the frequency of chromosome bridges. The bridges were observed in most of the studied oat amphiploids and parental species. Oats with AA and BB genomes have heterochromatic C-bands located at the telomeres (Fominaya et al. 1988b), whereas, in those with CC genomes, heterochromatin is more condensed and present at telomeres and in intercalary loci (Fominaya et al. 1988a; Jellen et al. 1993). In the studied material, three cases of DAPI knobs and AT-rich heterochromatin were detected on bridges in oats with AA, BB, and CC genomes, and probably, these knobs belong to C genome with more condensed heterochromatin. Data on genomic sets in the oat endosperm presented by Tomaszewska (2017) show that the tissue endosperm of the amphiploid *A. eriantha* × *A. sativa* has four doses of genome C; other amphiploids have two doses and *A. abyssinica* × *A. strigosa* none. The telomeric AT-heterochromatin in the last amphiploid (Fig. 1b) does not belong to genome C and does not form bridges. Tomaszewska (2017) proved that some chromosomes from A/D and C genomes were eliminated in the free-nuclear endosperm; therefore, the above estimation of endospermal genomic sets can vary. In a line of Triticale producing shrivelled seeds, Varghese and Lelley (1983) detected a high frequency of endosperm polyploid nuclei and a more than 18% reduction in the nuclei number. Bridges with and without heterochromatic bands were formed. In the opinion of these authors, it is not the heterochromatin but the sister chromatid exchange that may cause incorrect connections of chromosome ends and chromosome bridging, whereas the spindle anomaly can form aberrant nuclei. The latter is corroborated by multipolar anaphases observed in *Avena* endosperm (Tomaszewska 2017).

Kilian et al. (1998) proved that endosperm telomerase activity in barley and maize is distinctly lower than that in the embryo and can be responsible for dysfunction of telomeres, but their data relate to older, cellular tissue, and not to that of free-nuclear stage. The hypothesis about the correlation between poor telomeres and telomerase activity versus cytogenetic anomalies in endosperm should be checked. Our data show that both types of chromosomes, with and without telomeric AT-rich heterochromatin, can form bridges. Thus, Bennett’s hypothesis (Bennett 1977) on the correlation between telomeric heterochromatin and bridge formation in Triticale does not necessarily have to be valid for each grass taxonomic unit.

In oats, many evidenced cytogenetic disorders entail loss of many aberrant nuclei through the apoptosis-like process more common in amphiploids and less so in species. Such a cytogenetic behaviour causes not only a large genetic change in the syncytium, but also creates new spatial relationships for nuclei which still undergo karyokineses and for the development of cellular endosperm formed later. Thus, the tissue develops according to a new architectural pattern.

### Macrodisorders

Hybrid grass endosperm phenotypes are often similar to those obtained in the mutagenic process. For instance, in the interploidy 4*x* × 2*x Hordeum vulgare* hybrid endosperm, the central vacuole of the young caryopsis is free of starch granules and remains a-cellular (Håkansson 1953). When the synthesis of starch is sufficient, this vacuole is filled by starch granules, but persists as a-cellular. In cereals, the a-cellular domains, not always filled by starch, are evidenced in mutants, *sex* B9 in *Hordeum vulgare* (Bosnes et al. 1987) and *su se* in *Zea mays* (Harris and DeMason 1989). The lack of cellularisation is correlated with defects in the vacuolarisation of syncytial endosperm (Bosnes et al. 1992). In maize, an *endosperm defective1* mutant is affected in cellularisation due to the lack of a radial system of microtubules and fragmoplasts (Pignocchi et al. 2009).

A-cellular starchy domains, such as those in *Avena longiglumis* (Fig. 2a), have also been detected in the amphiploid *A. abyssinica* × *A. strigosa* (Kosina and Tomaszewska 2011) and in *Bromus secalinus, Avena strigosa, A. brevis*, and *Aegilops umbellulata* (R. Kosina, unpbl.).

In general, the endosperm structure studied in the caryopsis cross-section expresses cytokinetic and growth asymmetry between dorsal and ventral parts. For instance, in *Thinopyrum distichum*, the dorsal part has more cytokineses and more extensive elongation growth. Such a development also gives distinct cellular clones (Kosina 2012). As a rule, the elongation growth dominates in the centre of the caryopsis cross-section, while, in lateral parts, isodiametric cells are developed. However, long, cylindrical cells prevail on the total cross-section in *Avena barbata*, whereas isodiametric cells are the main component of starchy endosperm in the amphiploid *A. barbata* × *A. sativa* ssp. *nuda* (Kosina and Tomaszewska 2011). Any cytogenetic loss and the smaller number of functional nuclei preserved in the endosperm syncytium create a new spatial offer for free cellular growth. Then, many aleurone and also nucellar cells can grow into starchy tissue, and also large polyploid clones in starchy and aleurone endosperm can easily develop. Extensive ingrowths separate endospermal domains that can vary between each other with respect to cell structure and/or cell metabolism. Aleurone ingrowths have been described in the intergeneric amphiploids in the tribe Triticeae (Kosina and Tomaszewska 2010) and polyploid starchy clones in *Avena longiglumis* (Kosina and Tomaszewska 2011) and *Avena barbata* × *A. sativa* subsp. *nuda* amphiploid after 5-azaC treatment (Florek and Kosina 2017). Cells adjacent to such polyploid clones were apoptotically eliminated.

Endospermal slits, such as those in oats, and long aleurone cells expressing intrusive growth have also been documented in intergeneric amphiploids of the Triticeae tribe (Kosina and Tomaszewska 2010). In an *Avena barbata* × *A. sativa* ssp. *nuda* amphiploid with demethylated genomes by 5aza-C, especially deep aleurone ingrowths separated two or three large sectors (Florek and Kosina 2017). However, in F_1_ *Triticum* × *Elymus* hybrids, neighbouring starchy cell clones expressing two starch granule phenotypes were not separated by aleurone ingrowths and were evidently of somatic mutation origin (Ivanovskaya 1983).

### Aleurone layer

In oats, the multicellular aleurone layer is the result of additional periclinal divisions in cells expressed earlier as the phenotype with aleurone grains. In an experiment with various grasses, the number of cells in the aleurone layer was amplified by periclinal divisions after the local application of artificial external pressure on the young caryopsis (Kosina 2015). Thus, the aleurone layer may be called ‘an endosperm cambium’. Its external cells built on the preserved embryo sac wall are meristematic and not finally differentiated up to the last periclinal cytokineses. The level of amplification of such divisions can be attributed, inter alia, to the level of external pressure exerted by sclerified tissues of caryopsis and spikelet organs and the internal pressure of expanding the starchy tissue. However, the paternal genetic background of a multilayered aleurone has been observed in the recombinant variation of *Triticum* × *Thinopyrum distichum* amphiploids (Kosina and Tomaszewska 2012). In grass hybrids, the aleurone layer reveals a callus-like development near many empty spaces in the starchy tissue or near its looser structure. Such a type of aleurone has also been described in the amphiploid *Triticum timopheevii* × *Aegilops longissima* (Kosina et al. 2016). Sometimes, the unordered multicellular aleurone is composed of large, polyploidised cells; for example, in the amphiploid *Triticum turgidum* × *Thinopyrum distichum* (Kosina and Tomaszewska 2012) or *Leymus racemosus* (Kosina et al. 2015).

In addition, apoptotic elimination of some aleurone cells has been observed in the amphiploid *Triticum turgidum* ssp. *dicoccum* × *Aegilops tauschii* (Kosina et al. 2015). Such a disappearance of the aleurone cells can create false evidence in a ripe caryopsis, that the aleurone layer is not locally developed. Two states of the tissue, its real lack versus apoptotic disappearance, should be distinguished in any study. The real lack of aleurone cells is documented in Fig. 3f. Periclinally undivided, large starch cells can persist between cells of the aleurone layer from the early stages of development. Thus, the lack of periclinal division does not allow the expression of the aleurone phenotype. Hence, this suggests that the starch phenotype is equivalent in time to the aleurone phenotype. Such a conclusion is opposite to Becraft & Yi’s opinion related to maize aleurone development explaining the redifferentiation of starchy cells from the aleurone phenotype (Becraft and Yi 2011). In cereals, aleurone cells are usually detected by various markers several days after pollination, when the endosperm is highly cellular (Morrison et al. 1975; Becraft and Asuncion-Crabb 2000; Costa et al. 2003; Gruis et al. 2006). Two main characteristics should be considered as indicators of the aleurone phenotype: storage of aleurone grains and enzymatic function during germination. Other markers can identify the external position of the cell and its special walls, but its final phenotype can differ from the aleurone one. Thus, two basic questions should be put here: whether, at the earliest stages of the syncytium cellularisation, the external cell differentiates into the aleurone unit and its sister cell redifferentiates into a starchy one after periclinal division; or whether the external cell persists undifferentiated and its sister cell, after the last periclinal division, differentiates into a starchy one? The latter suggestion means that the external cell maintains its meristematic status for a long time, and is undifferentiated. The last periclinal division can release its aleurone nature. It is possible that the assimilate supply determines the developmental choice: to store starch or to divide. Poor assimilate supply causes poor starch storage, and this is observed in outer parts of the endosperm (Kosina 2012, 2014). The aleurone cells never store starch.

An additional phenotype of endosperm cells, which most often form a subaleurone layer and store high protein (HP), can also be expressed within the aleurone layer (Fig. 3c). Its outer cells, created by the last periclinal cytokineses, are not changed into aleurone ones. Such a development proves that, in this case, the HP phenotype is genetically stable. Therefore, the HP clone is formed earlier via a somatic mutation, which may be similar to SCO in Fig. 4.

The aleurone cells developed within the starchy endosperm (Fig. 3d) can be formed by ingrowths of the outer aleurone layer, such as that evidenced in maize by Olsen (2004), or they can be a cell group autonomously differentiated as the aleurone phenotype. Costa et al. (2003) evidenced the aleurone cells inside the starchy tissue in the *glo1-1* maize mutant. Such a picture is corroborated by a single aleurone cell detected inside the starchy tissue in *Brachypodium distachyon* which is not spatially connected with the outer aleurone layer. This cell shows a sister connection with an adjacent starch cell and can show the somatic crossing-over origin of an aleurone unit inside the starchy endosperm (R. Kosina, unpbl.). Somatic crossing-over causing the formation of new aleurone and starch phenotypes is documented in Fig. 4e, f and in the intergeneric amphiploids in the tribe Triticeae (Kosina and Tomaszewska 2010). A somatic crossing–over event should always be checked for the development of various phenotypes of the sister aleurone cells. For instance, the aleurone cells can be expressed in the form of various phenotypes (see Fig. 4, and Kosina et al. 2016). Some of these are rare; for instance, a ‘sclereid-like’ cell in oat (Florek and Kosina 2017) or undifferentiated aleurone cells in *nkd* mutants in maize (Becraft 2007).

Becraft and Asuncion-Crab (2000) concluded for the maize endosperm that the fate of an aleurone phenotype is determined by its outer position. They also documented that, in *dek 1* mutants, differentiation of the aleurone layer is blocked, but peripheral starchy cells at a transposon-induced reversion can redifferentiate into aleurone units. Indeed, the specificity of the external position of the aleurone cell is easy to understand, because this cell is developed, ab initio, on the old wall of an embryo sac, which is different from the walls of other cells, which develop inward later. Becraft (2001) points out that after the first periclinal cytokinesis in the peripheral layer in maize, the fate of the new cells is plastic during endosperm development. In wheat, Morrison et al. (1975) proved that the strong markers, such as pre-globoids and pre-aleurone grains, evidencing the aleurone phenotype of the peripheral layer, were observed several days after pollination. A negative gradient between the starch granule size and their amount per cell versus the amount of stored protein has been documented in *Thinopyrum distichum* between the inner and outer parts of the endosperm (Kosina 2012). The peripheral cells situated further from the caryopsis transfer system receive less assimilates and store proportionally more protein (Kosina 2014). Thus, two stimuli assimilate support and the last periclinal division, beyond the genetic background, would be considered as promoting the peripheral cell to express an aleurone nature.

The multilayered aleurone expressed in the maize *thick aleurone1* (*thk1*) mutant is probably created by chromosome deletion. Such an aleurone trait has a quantitative genetic background (Yi et al. 2011). It is likely that, in oats, chromosomal disorders and eliminations allow similar endosperm development.

Analyses of the amphiploid aleurone layer in *Triticum* × *Aegilops* (Kosina et al. 2016) and *Elymus canadensis* × *Pseudoroegneria libanotica* (Kosina and Tomaszewska 2010) have shown a mosaic of aleurone globoid synthesis. In oats, a higher number of larger globoids were synthesised in the dark aleurone cells. Such a mosaic structure of the aleurone layer may result from impaired transport of assimilates in the developing caryopsis. Here, the distance between the aleurone cells and vascular tissues can determine the support of globoid components. In cereals, some mutants with numerous small globoids are known; for example, a *low phytic acid* mutant has been reported for *Oryza sativa* (Liu et al. 2004), *Hordeum vulgare* (Ockenden et al. 2001), and *Triticum eastivum* (Joyce et al. 2005).

The other mosaic of aleurone cells with large vacuoles occurring only in the studied oat species indicates the metabolic difference between parts of a single aleurone layer as well as between species and their hybrid progeny. In the aleurone cells of barley, two types of vacuoles with different functions have been identified: one large, for protein storage; one small, with a similar function to that of lysosome (Swanson et al. 1998). If large vacuoles in oat aleurone cells are homologous to those in barley, they express unfinished metabolism of aleurone grains related to the dehydratation of protein. Similar vacuolated cells have been noted for *Leymus arenarius* (Kosina et al. 2016). The large vacuoles in barley under germination are irregular and participate in the process of autolysis rather than that of apoptosis (Fath et al. 2000). In oats, such vacuoles (Fig. 4a) are also irregular and can mark a pre-germination process.

The development of clones of small or large oat aleurone cells, expressing the changed length of the cell cycle, relates well to the development of similar clones in cultivars of *Hordeum vulgare*, that arise with an increased frequency after X-ray irradiation (Kosina 2007). The clones of small cells in a *dil1-2* maize mutant were formed into a callus-like body, after anticlinal plus periclinal cytokineses (Lid et al. 2004). Highly polyploid, giant aleurone cells have been observed in other grasses (Ivanovskaya 1983; Kosina et al. 2016) and this corroborates the belief that polyploidisation is a common phenomenon in grass endosperm (Becraft and Gutierrez-Marcos 2012). However, in species and hybrid oat derivatives studied here, it is not triploid but diploid nuclei that dominate in the endospermal syncytium. Thus, the level of ploidy and genomic interactions is different from those established in other cereals with 3*n* endosperm (Tomaszewska 2017).

The development of single or large complexes of starchy cells in the aleurone layer occurred at a higher frequency in oat amphiploids than in species. A similar development has been observed in maize (Lid et al. 2004) and barley (Olsen et al. 2008) mutants. Redifferentiation of starchy cells into aleurone and vice versa detected in *defective kernel1* (*dek1*) maize mutants points to the developmental plasticity of the aleurone layer (Gruis et al. 2006). The starchy and protein cell mosaics in the aleurone layer also occur in the *crinkly4* maize mutants (Becraft and Yi 2011). The last mitotic division determines the identity of aleurone cells in the endosperm and its lack results in a starch unit in the aleurone layer (see Fig. 3f). The HP phenotype formed within the aleurone layer and expressing the last periclinal cytokineses shows that the role of such divisions for determination of the aleurone layer is limited in the mutated within the endosperm tissue cell clones.

Metabolites not used for cytokinesis of a pre-aleurone cell serve for DNA replication and growth of the starchy cell. Therefore, starchy cells in the aleurone layer are most often large and polyploid. However, aleurone cells can also develop inside the starchy endosperm (Costa et al. 2003). Data for *Brachypodium distachyon* show that such a development can be realised via somatic crossing-over (R. Kosina, unpbl.). Analyses of cell clones in the aleurone layer in *Avena fatua* × *A. sterilis* and other grass amphiploids prove that somatic crossing-over can result in starchy cells or other aleurone phenotypes in the layer (Kosina and Tomaszewska 2010, 2011).

## Concluding remarks

The presented data allow the following statements on oat endosperm. Cytogenetic anomalies in a free-nuclear syncytium occur more frequently and express larger variability in the hybrid progeny than in pure species. Chromosomes or their fragments such as micronuclei and abnormal nuclei are eliminated through apoptosis-like (condensation and fragmentation) process. Bridges are created by chromosomes both with and without telomeric AT-heterochromatin. The change of the genetic background by the BFB cycle is possible. The cytogenetic disorders provide a new architectural space for further clonal development of the endosperm. Large structurally and metabolically different sectors are formed within the endosperm. The elongation and intrusive growth of aleurone cells and/or nucellar tissue delineate these sectors. The multicellular aleurone is formed by periclinal cytokineses or by divisions such as that in a callus. Isolated groups of aleurone cells can develop inside the starchy tissue. The starchy cells within the aleurone layer are most often polyploid. The lack of a periclinal division that finally separates the aleurone layer is typical for these polyploid cells. The aleurone layer takes the form of cellular mosaics with changes related to the length of cell cycle or metabolism. Mitotic crossing-over occurs at the last anticlinal karyokineses in the layer and this event is one of the factors responsible for the layer variability. Within the aleurone layer, HP cell clones can also be established due to an earlier somatic mutation. The developmental events in oat endosperm are similar to those that occurred under the control of several genes (*dek1, crinkly4, glo1*, and *sal1*) documented in other cereals.

### Author contribution statement

PT designed and conducted all cytogenetic and microstructural analyses, interpreted results, and prepared tables and figures. RK provided research idea, supervised the experiments and research documentation, performed numerical analyses, and wrote the article.
