# Correction to: Instability of endosperm development in amphiploids and their parental species in the genus *Avena* L.

**DOI:** 10.1007/s00299-018-2337-y

**Published:** 2018-08-28

**Authors:** Paulina Tomaszewska, Romuald Kosina

**Affiliations:** 1 Institute of Experimental Biology, University of Wroclaw, Kanonia 6/8, 50-328 Wroclaw, Poland; 2 Institute of Environmental Biology, University of Wroclaw, Przybyszewskiego 63, 51-148 Wroclaw, Poland

## Correction to: Plant Cell Reports (2018) 37:1145–1158 10.1007/s00299-018-2301-x

The article ‘Instability of endosperm development in amphiploids and their parental species in the genus *Avena* L.’, written by Paulina Tomaszewska and Romuald Kosina, was originally published electronically on the publisher’s internet portal on 22 May with an incorrect copyright line. As the article was due to be published with Open Choice, the copyright of the article changed on 29 August 2018 to © The Author(s) 2018 and the article is forthwith distributed under the terms of the Creative Commons Attribution 4.0 International License (http://creativecommons.org/licenses/by/4.0), which permits use, duplication, adaptation, distribution and reproduction in any medium or format, as long as you give appropriate credit to the original author(s) and the source, provide a link to the Creative Commons license and indicate if changes were made.

The original article has been corrected.

